# Recent advances in hydrophilic modification and performance of polyethersulfone (PES) membrane *via* additive blending

**DOI:** 10.1039/c8ra03296c

**Published:** 2018-06-20

**Authors:** Tunmise Ayode Otitoju, Abdul Latif Ahmad, Boon Seng Ooi

**Affiliations:** School of Chemical Engineering, Engineering Campus, Universiti Sains Malaysia 14300 Nibong Tebal Penang Malaysia chlatif@usm.my +60-45941013 +60-45995999

## Abstract

The blending of additives in the polyethersulfone (PES) matrix is an important approach in the membrane industry to reduce membrane hydrophobicity and improve the performance (flux, solute rejection, and reduction of fouling). Several (hydrophilic) modifications of the PES membrane have been developed. Given the importance of the hydrophilic modification methods for PES membranes and their applications, we decided to dedicate this review solely to this topic. The types of additives embedded into the PES matrix can be divided into two main categories: (i) polymers and (ii) inorganic nanoparticles (NPs). The introduced polymers include polyvinylpyrrolidone, chitosan, polyamide, polyethylene oxide, and polyethylene glycol. The introduced nanoparticles discussed include titanium, iron, aluminum, silver, zirconium, silica, magnesium based NPs, carbon, and halloysite nanotubes. In addition, the applications of hydrophilic PES membranes are also reviewed. Reviewing the research progress in the hydrophilic modification of PES membranes is necessary and imperative to provide more insights for their future development and perhaps to open the door to extend their applications to other more challenging areas.

## Abstract 2

The blending of additives in the polyethersulfone (PES) matrix is an important approach in the membrane industry to reduce membrane hydrophobicity and improve the performance (flux, solute rejection, and reduction of fouling).

## Graphical abstract

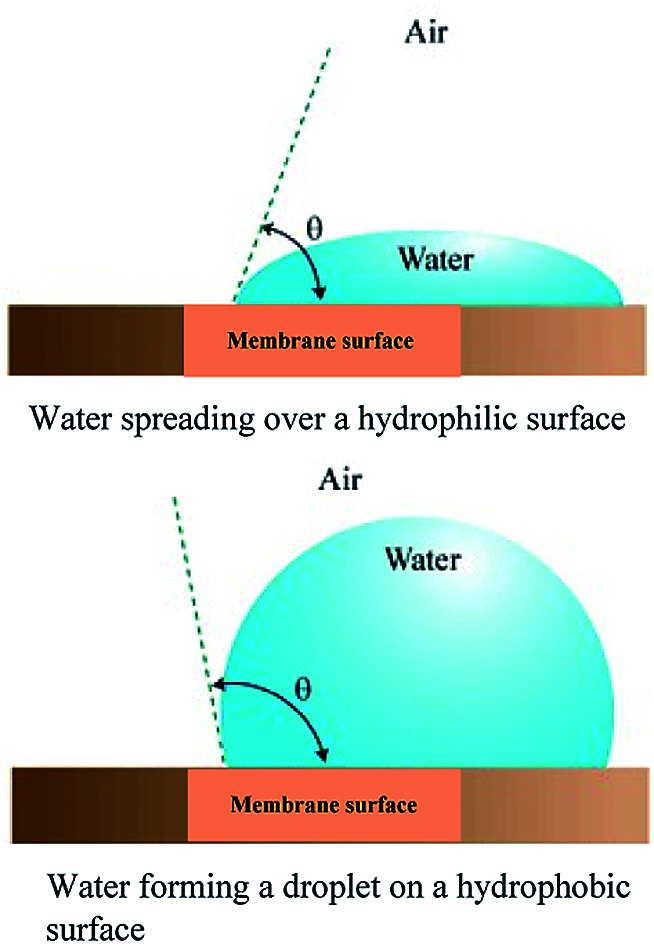

## Introduction

Polyethersulfone (PES) is a recognized polymeric material, which is widely employed in the fabrication of membranes for various applications. Due to its high glass transition temperature (225 °C), and amorphous and transparent properties, PES possesses a high mechanical and hydrolytic stability, thermal and chemical resistance, and outstanding oxidative characteristics,^1^ making it ideal for the preparation of asymmetric membranes with different surfaces and pore sizes.^1–3^ Asymmetric PES membranes are generally prepared *via* a phase-separation method. The final membrane properties and performance are influenced by the composition (additives, concentration, and solvent), temperature of the doping solution, the non-solvent or the mixture of non-solvents, and the coagulation bath or the environment.^4^ The risk of the fouling effect due to the high hydrophobicity of PES, especially in protein-contacting applications and aqueous filtrations, limits their wide applications.^2,3^ Numerous research studies have reported efforts to enhance the hydrophilicity of the PES membrane surface.^1,5^

Basically, the water contact angle (WCA) formed between the membrane–liquid boundary and liquid–gas tangent is generally employed to evaluate the hydrophilic properties of the membrane.^6^ Commercial PES membranes are hydrophobic in nature with high mechanical, chemical, and thermal stability.^7^ Usually, these membranes possesses high WCA values and are prone to solute adsorption from various feed streams. It has been well documented that membranes with hydrophilic surfaces are less prone to the fouling effect with microorganisms and organic substances due to: (i) a decreased interaction between the membrane surface and foulant, and (ii) no interaction of hydrogen bonds in the boundary layer between water and the membrane interface.^8–10^ The repulsion of water molecules away from the surface of the hydrophobic PES membrane is a spontaneous process with increasing entropy, and therefore foulant molecules have a tendency to dominate the boundary layer and adsorb onto the PES membrane surface. However, a modified PES membrane with a hydrophilic chain and high surface tension can enhance the formation of hydrogen bonds with the surrounding water molecules. This hydrogen bonding can reduce or prevent the adhesion of foulants on the surface of the PES membrane.^11,12^ The membrane WCA is related to the zeta potential, surface roughness, and functional groups.^13,14^Ref. 15 and 16 demonstrated that an improved membrane hydrophilicity can be favored by increasing the density of the surface hydrophilic-group, including –NH_2_ and –OH.

Numerous studies on PES membranes have been carried out with the aim to enhance the hydrophilicity and performance, including through an improvement in their preparation process (blending) and by surface modification of the nascent membranes. In surface modification, a hydrophilic layer is formed on the existing PES membrane surface, which can then aid the prevention of contact between the solute and membrane surface, thus reducing the membrane fouling effect. The surface modification can be classified based on two categories, namely chemical or physical modification. In chemical modification, the PES membrane surface is modified through covalent bonding interactions. In this procedure, PES chains are first activated by chemical reaction, followed by the grafting with hydrophilic additives. The use of surface modification may render the hydrophilicity permanent, but may, however, lead to degradation of the PES chains on the membrane surface.^17^ In practice, these methods usually require caustic chemicals, which limits their wide use and long-term stability in membrane applications. Physical modification signifies that the hydrophilic modifiers exist on the PES membrane surface *via* physical interaction. Here, the blending approach is a versatile and convenient procedure under mild conditions to enhance the hydrophilicity and performance of PES membranes.^18,19^ Blending is a process in which two (or more) inorganic and/or organic materials are physically mixed to obtain the required properties on the membrane. This introduction can be achieved by adding polymer material and inorganic nanofillers into the casting solution. Table 1 presents the advantages and disadvantages of both approaches. Since most of these additives are hydrophilic in nature, they are able to increase the hydrophilicity of the resulting membranes and thus can reduce the fouling effects. Other advantages of blending with hydrophilic additives include an increase in the water flux (WF) due to the enlarged effective membrane surface area and the introduction of additional functional groups.^20^

**Table 1:** Advantages and disadvantages of incorporating polymer and inorganic additives in the PES membrane matrix

Membrane synthesis approach	Advantages	Disadvantages
Blending with a polymer	(a) Miscibility in common solvents	(a) Tendency toward physical and/or chemical aging
	(b) Flexible to incorporate	(b) Leaching during the preparation and operation process, which may reduce the efficiency
		(c) Poor compatibility in the polymer matrix
Blending with an inorganic material	(a) High chemical resistance	(a) More expensive than equivalent polymeric ones
	(b) Improve thermal stability	(b) Defect-free commercial-scale inorganic PES membranes are difficult to manufacture
	(c) The resultant membrane combines the advantages of the organic and inorganic parts	(c) Stability of the doped form is a big issue due to its nano-size
	(d) Can be easily incorporated	(d) Very expensive formulations
	(e) Provides an enhanced surface that allows multiple functional groups to be added on the membrane surface	(e) Non-uniform dispersion of NPs in the polymer matrix
		(f) Aggregation phenomenon
		(g) Weak interaction with the polymer matrix
		(h) Leaching of NPs during the operation process
		(i) Uncontrollable pore size
		(j) Poor dissolution in various organic solvents

### Embedding polymer materials

In this approach, hydrophilic organic polymers are dissolved in PES solution. The materials most commonly used include polyvinyl pyrrolidone (PVP), chitosan (CS), polyamide, polyethylene oxide (PEO), and polyethylene glycol (PEG) derivatives due to their reasonable price and high compatibility with PES.^21^Table 2 shows the progress reported in recent studies on polymeric addition in PES membranes.

**Table 2:** Progress of recent studies for the fabrication of hydrophilic PES–polymer blend membranes

Additive	Additive loadings (wt%)	Hydrophilic change (°)	Ref.
PEG	2	∼70 to ∼57	22
PEG/PVP	—	85 to 59	23
PVP 40K	4	71 to 47	24
PVP	5	∼63 to ∼56	25
P31R1	5	∼63 to 44	25
PVP	2	∼76 to ∼71	26
T904	5	∼63 to ∼52	25
PVP	2	∼76 to ∼71	27
PA-6	2	∼76 to 68	26
PVP	10	70 to 51	28
NPhthCs	0.9	61 to 56	29

One general issue of blending with a polymer is the elution of this polymer and poor compatibility with the PES matrix.^17^ To address this issue, some researchers have looked into the use of amphiphilic copolymers as well as amphiphilic copolymers containing PES chains with hydrophobic parts as modifiers. Amphiphilic modifiers contain hydrophilic and hydrophobic properties, which means they are able to interact with the hydrophobic PES polymer, which is totally insoluble in water, and are also able to interact with hydrophobic PES polymers. The hydrophobic chains guarantees the compatibility with the host PES polymer, while the hydrophilic chains are enriched onto the membrane pore during phase inversion due to a segregation effect, thus, providing a high coverage of hydrated side chains anchored by a hydrophobic backbone entangled with the PES bulk that is water insoluble.^30,31^ Moreover, by controlling the ratio of hydrophobicity and enhancing the hydrophilicity during the membrane casting, desirable membrane performance properties can be achieved, such as a higher solute rejection, fouling resistance, and permeability.^32^Table 3 show a summary of hydrophilic PES-amphiphilic copolymer blend membranes.

**Table 3:** Progress in recent studies in the fabrication of hydrophilic PES-amphiphilic copolymer blend membranes

Additive	Synthesis of the additive	Additive loadings (wt%)	Hydrophilic change (°)	Ref.
PDMAEMA	RAFT	20	∼84 to 56	33
PNIPAAm	RAFT	20	∼84 to ∼71	33
F127-*b*-PDMAEMA	ATRP	15	72 to 53	34
PSf-*g*-POEM	ATRP	5	85 to 52	35
PS-*b*-PAA	Free radical polymerization	—	∼70 to ∼50	36
MF-*g*-PEG_6k_	Etherification	0.36	—	37
PMAAn–F127–PMAA	Free radical	1.92^a^	∼56 to 39	38
PSA-PVP	Condensation reaction of 5, 5′-thiobis (4-(3-nitrophenyl) thiazol-2- amine) in the presence of terephthalic acid	1	∼76 to 68	27
PVP-*b*-PMMA-*b*-PVP	RAFT	5	73 to 60	39
PES-*g*-PSBMA	RAFT and quaternization	15	90 to 60	40

a Unit in grams

Generally, it has been well documented that these copolymers have better compatibility with the PES bulk, and could be used as modifiers to enhance the hydrophilicity, antifouling properties, and performance of PES membranes.^41–44^

### Embedding inorganic materials

Apart from introducing polymers and copolymers, inorganic NPs are another promising modifier. The addition of inorganic NPs with the PES matrix has become an attractive approach for the fabrication of polymeric membranes and has captured much attention in recent times.^45–50^ Much of the bulk of the research has been carried out on the preparation of composite PES–inorganic membranes by the addition of inorganic NPs. For instance, the presence of dispersed inorganic NPs in the membrane matrix has been reported to improve the membrane performance and properties, particularly by: (a) increasing the permeability due to the larger effective membrane surface area of NPs; (b) inducing a membrane with the functional properties of the nanomaterials;^20^ (c) enhancing the mass transfer in the membrane pre-evaporation process;^51^ (d) improving a membrane's hydrophilicity as well as fouling resistance properties;^46,52^ (e) improving the thermal and mechanical properties.^53–58^ To date, many types of inorganic materials have been incorporated as additives in the PES matrix, including titanium dioxide, silicon dioxide, carbon nanotubes, halloysite nanotubes, manganese oxide, cellulose nanocrystals, graphene oxide, silver NPs, zirconia, zinc oxide, alumina, and metal–organic frameworks. However, there are two ways to introduce these NPs into the PES membrane during the preparation process: blending them in a coagulation bath or in the polymer solution. Compared to the blending of nanofillers in a coagulation bath, blending the nanoparticles in the polymer solution has been the dominant method. The discussion below introduces the incorporation of inorganic additives in the PES matrix.

### Embedding titanium dioxide NPs

Titanium dioxide (TiO_2_) has been the major focus of quite a significant number of studies in recent and past years, due to its photocatalytic effects, which aid in killing bacteria and decomposing organic chemicals, relative cheapness, chemical stability, optical property, and non-toxicity.^49,59–65^ As one of the most investigated NPs, when TiO_2_ NPs are dispersed in the PES matrix, the membrane hydrophilicity and antifouling ability can be enhanced. On this basis, lots of effort has been devoted to investigating the effect of TiO_2_ NPs to improve the PES membrane hydrophilicity. For instance, ref. 66 introduced TiO_2_ to produce PES–TiO_2_ membranes and found that modification with 0.2 wt% led to an improved hydrophilicity as WCA decreased from 75.2° to 66.4°. Another study by ref. 67 showed a decrease in WCA from 72.2° to 57.4° when the content of TiO_2_ was 1 wt% in the PES matrix. The result by ref. 68 showed an improved hydrophilicity of 54.2° when the content of NPs was 0.1 wt% as compared to an unmodified membrane (65.5°). Ref. 69 observed a significant reduction in WCA from 71.9° to 59.6° when the content of TiO_2_ NPs was 2 wt%. Ref. 70 also reported a PES/TiO_2_ composite membrane that resulted in an enhanced hydrophilicity of 44.1° upon the introduction of 0.4 wt% TiO_2_ as compared to the neat membrane of 52.3°. Ref. 71 introduced a mechanically modified TiO_2_ into the PES matrix and observed a reduction in WCA from 64° to 56° when the concentration of the modified NPs was increased to 2 wt%. Furthermore, the hydrophilicity was improved to 50° when mechanically and chemically modified TiO_2_ was introduced. This result was similar to that reported in the study by ref. 69, who observed an improved hydrophilicity from 71.9° to 62.3° when the content of a mechanically and chemically modified TiO_2_ was 2 wt%.

### Embedding silica (SiO_2_) NPs

**Fig. 1:**
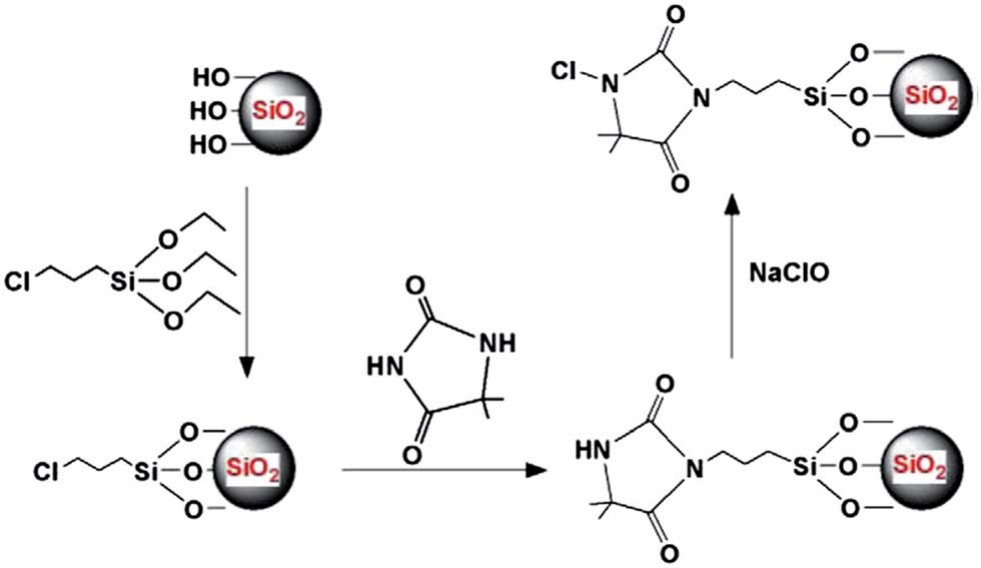
Basic principles of the reactions with modified SiO_2_.^77^

The addition of SiO_2_ NPs has been investigated intensively and proven ideal as an additive for PES membranes due to their many useful properties, such as fine suspendability in aqueous solution, relatively environmentally inert, being thermally and chemically stable with a large surface area (SA), and highly miscible.^72^ A significant number of works have been reported on their addition into the PES matrix and they have been found to be a promising additive for enhanced hydrophilic PES membranes. For example, ref. 73 introduced a SiO_2_/PES membrane, which showed a significant reduction in WCA from 78.6° to 58.1° when the SiO_2_ content was 2 wt% in the matrix. Ref. 74 prepared a PES nano-SiO_2_ membrane by introducing monodisperse silica spheres in the PES matrix, which led to an improvement in hydrophilicity with WCA decreasing from 52.4° to 45.7° when the content of NPs was 0.3 wt%. Ref. 75 synthesized a series of amine-functionalized mesostructured silica (SBA-15) particles and then incorporated these in the PES matrix. The synthesis of the different organically functionalized SBA-15 particles was similar to that for the conventional SBA-15 except for the addition of a certain amount of selected organosilanes 1 h after adding tetraethyl orthosilicate (TEOS). In the case of the amino-functionalized silica materials, [3-(2-aminoethylamino) propyl] trimethoxysilane (AEAPTMS) and (3-aminopropyl) trimethoxysilane (APTMS) were used as an organosilane with an organosilane/TEOS molar ratio of 15%. In the case of the carboxylic-functionalized silica materials, carboxyethylsilanetriol sodium salt (CES) was used as a carboxylic group source with organosilane/TEOS molar ratios of 15% and 30%. The WCA of the neat membrane and the PES/mesostructured SBA-15 were 70.3° and 64.7°, respectively. However, upon the introduction of 0.6 wt% SBA-15/CES-15, SBA-15/AEAPTMS-15, and SBA-15-APTMS-15, the WCA decreases to 63°, 61.7°, 58.1°, and 55°, respectively. Ref. 76 prepared a hydrophilic hollow mesoporous silica sphere (HMSS) prepared *via* a surfactant-assembly sol–gel route, which was then blended into PES membranes to fabricate a mixed matrix membrane. The WCA of the pure PES membrane was 76.8°, indicating a strong intrinsic hydrophobicity of the pure PES membrane. However, with the increase in HMSS loading (up to 1.5%), the WCA of the PES composite membrane decreased to 63.8°. Ref. 77 embedded a *N*-halamine-modified SiO_2_ in the PES matrix to prepare SiO_2_@N-Halamine/PES MMM. Modified SiO_2_ NPs grafted with *N*-halamine were obtained *via* a three-step reaction process (Fig. 1). Their result showed an improved hydrophilicity with a WCA of 70.6° using 5 wt% of modified NPs, which was lower than the neat membrane WCA of 90.7°.

### Embedding zinc oxide NPs

**Fig. 2:**
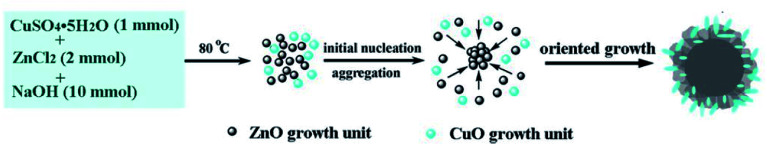
Schematic illustration of the formation process of CZN.^87^

Zinc oxide (ZnO) is another compound that has excellent electrical, optical, chemical, and mechanical properties, including antimicrobial activity.^78–80^ With its low cost and increased surface-to-volume ratio, ZnO is a potential NP that could meet the demand for the fabrication of a lower-cost and efficient membrane. For example, ref. 81 prepared a PES ZnO HF membrane by introducing ZnO NPs in the PES matrix, which led to an improvement in the hydrophilicity, with WCA decreasing from 70° to 58° when the content of NPs was 3.62 wt%. Ref. 82 observed a significant reduction in WCA from 79.92° to 62.92° when the content of ZnO NPs was 0.3 wt%. Ref. 83 also observed a significant reduction in WCA from 71.4° to 57.7° when the content of ZnO NPs was 10 wt%. Ref. 84 prepared and compared two nano-ZnO (ZnO NPs and ZnO nanorod) and then introduced them in the PES matrix. The ZnO nanoparticles were synthesized by co-precipitation technique, while ZnO nanorods were synthesized by the sol–gel method. In their study, the hydrophilicity of the ZnO-blended membranes rose to 60° at 0.1 wt% and reached 54° when the ZnO NPs were replaced with ZnO nanorods as compared to the neat membrane of 77.9°. Ref. 85 synthesized a chitosan (CS)-modified ZnO NPs using chemical precipitation prior to doping in the PES matrix to prepare a PES/CS-ZnO NPs membrane, which resulted to an improved hydrophilicity with the WCA declining from 60.73° for the neat membrane to 40.33° when the content of CS-ZnO was 15 wt%. Ref. 86 presented PES ultrafiltration membranes blended with different contents of the CuO/ZnO nanocomposite (CZN). In their study, CZN was prepared through a facile one-step homogeneous co-precipitation method at a low temperature (Fig. 2). Their results showed an improve hydrophilicity of 65.5° against 70.2° for the neat membrane at an optimal content of CZN (0.2 wt%).

### Embedding zirconium dioxide NPs

Zirconium dioxide (ZrO_2_), or zirconia, is a white crystalline oxide of zirconium with excellent chemical stability, melting point, good mechanical properties, and strong anti-corrosion. Zirconia membranes are known to be chemically more stable than alumina and titania PES membranes, and are more suitable for liquid phase applications under harsh conditions.^88^ For instance, ref. 70 presented ZrO_2_-entrapped membranes, which showed a slight reduction in WCA from 52.23° to 48.86° when the content of NPs was 0.4 wt%. In a study by ref. 89, hydrous ZrO_2_ sol was synthesized by the addition of an anion-exchange resin in *N*,*N*-dimethylformamide solvent containing zirconyl chloride and then doped in the PES matrix to prepare a ZrO_2_/PES composite membrane, which significantly led to a significant reduction in WCA from 73.6° to 52.3° at an optimal content of 1 wt%.

### Embedding aluminum oxide NPs

Similar to other metal oxide NPs, aluminum oxide (Al_2_O_3_) NPs have attracted great attention in membrane technology for the development of nanocomposite (NC) PES membranes with enhanced properties. Ref. 90 introduced Al_2_O_3_ to produce PES/Al_2_O_3_ membranes and found that modification with 0.1 wt% led to an improved hydrophilicity as the WCA decreased from 74.1° to 64.3°. Another study by ref. 70 showed a decrease in WCA from 52.3° to 37.8° when the content of Al_2_O_3_ was 0.4 wt% in the PES matrix.

### Embedding iron oxide-based nanoparticles

**Fig. 3:**
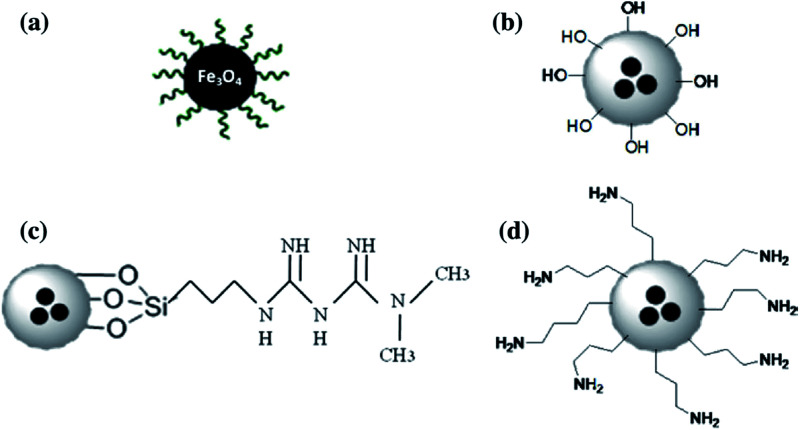
Schematic of (a) Fe_3_O_4_ treated with trisodium citrate, (b) Fe_3_O_4_/SiO_2_, (c) Fe_3_O_4_/SiO_2_-Met, and (d) Fe_3_O_4_/SiO_2_-amine NPs.^92^

The unique features of iron oxide-based nanoparticles (Fe-NPs), mainly magnetite (Fe_3_O_4_), have encouraged many researchers to investigate these engineered magnetic NPs in the synthesis of PES composite membranes. The addition of Fe-NPs has been extensively introduced in the PES matrix to produce composite membranes. For instance, ref. 91 prepared a PES/Fe-NP HF membrane by introducing magnetite NPs in the PES matrix, which led to an improvement in hydrophilicity with the WCA decreasing from 62.22° to 49.27° when the content of NPs was 2 wt%. Ref. 92 also presented four sets of PES/NC membranes, such as trisodium citrate-treated Fe_3_O_4_, Fe_3_O_4_/SiO_2_, Fe_3_O_4_/SiO_2_-amine, and Fe_3_O_4_/SiO_2_-metformine (Met). Fe_3_O_4_/SiO_2_ was prepared by the Stober method, while the surface functionalization of Fe_3_O_4_/SiO_2_ NP was achieved by using APTES as a silylation agent (Fig. 3). The WCA of the neat membrane was 78°. However, upon the introduction of 0.1 wt% Fe_3_O_4_/SiO_2_-amine, 0.1 wt% trisodium citrate-treated Fe_3_O_4_, 0.1 wt% Fe_3_O_4_/SiO_2_-Met, or 0.1 wt% Fe_3_O_4_/SiO_2_, the WCA decreased to 75°, 72°, 69°, or 67°, respectively.

**Fig. 4:**
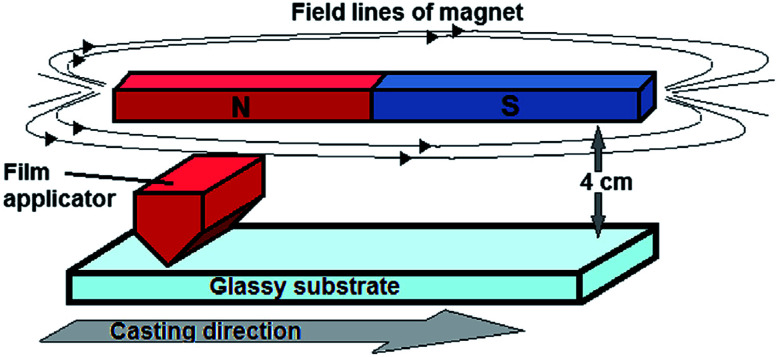
Scheme of casting under a magnetic field.^93^

Ref. 93 reported six sets of PES/NC membranes, such as magnetic-treated Fe_3_O_4_ (m-Fe_3_O_4_), magnetic-treated polyaniline-coated Fe_3_O_4_ (m-PANI/Fe_3_O_4_), magnetic-treated Fe_3_O_4_-coated multi-walled carbon nanotubes (m-MWCNT/Fe_3_O_4_), untreated Fe_3_O_4_, PANI-coated Fe_3_O_4_ (PANI/Fe_3_O_4_), and Fe_3_O_4_-coated MWCNT (MWCNT/Fe_3_O_4_) membranes. For magnetic field induced casting, the casting was carried out under a magnetic field (0.1 Tesla) at a distance of 4 cm (Fig. 4). The WCA of the neat membrane was 71.45°. However, upon the introduction of 0.1 wt% MWCNT/Fe_3_O_4_, 0.1 wt% m-MWCNT/Fe_3_O_4_, 0.1 wt% untreated Fe_3_O_4_, 0.1 wt% m-Fe_3_O_4,_ 0.1 wt% PANI/Fe_3_O_4_, or 0.1 wt% m-PANI/Fe_3_O_4_, the WCA decreased to 67.06°, 60.24°, 56.16°, 53.38°, 51.53°, or 51.12°, respectively.

### Embedding silver nanoparticles

**Fig. 5:**
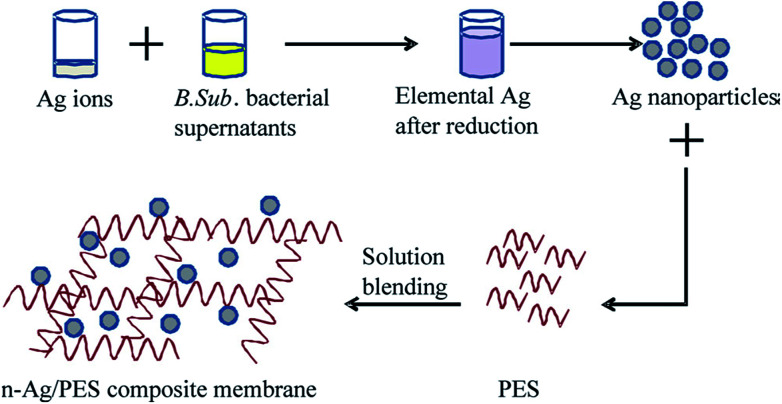
Schematic representation of the preparation of PES/*n*-Ag composite membranes.^99^

Within the wide range of commercially available nanoscale materials, silver nanoparticles (AgNPs) have also received a great deal of attention. AgNPs have unique properties (such as extremely large surface-to-volume ratio, antimicrobial, optical, and electrical properties), making the NP able to serve as a sustained local supply of Ag^+^ ions in membranes, and so it can prevent bacterial and solute adhesion onto the membrane surface.^94–96^ Several preparation techniques have been reported for the synthesis of silver NPs; notable examples include photochemical methods, gamma irradiation, laser ablation, microwave processing, electron irradiation, biological synthetic methods, and chemical reduction.^95^ The effects of AgNPs on the final membrane hydrophilicity have been investigated in many studies. A study by ref. ^97^ showed a decrease in WCA from 71° to 41° when the content of AgNP was 0.03 wt% in the PES matrix. Ref. 98 fabricated antibacterial PES/Ag nanocomposite membranes and reported an improvement in surface hydrophilicity from 59.85° to 40.29° when the AgNP concentration was 0.5 wt%. In another study by ref. 99, *n*-Ag NPs were prepared by a bacteria-mediated biosynthesis method (Fig. 5) and then introduced to produce the resulting composite membrane. The resulting PES membrane showed an improved hydrophilicity of 65° upon doping with 1.5 wt% NPs as compared to the neat membrane showing a value of 88°. This was associated as being due to the higher affinity or intrinsic nature of *n*-Ag with the water molecules.

### Embedding graphene oxide NPs

Graphene oxide (GO), a two-dimensional carbon material, has received tremendous attention due to the presence of abundant O_2_-containing functional groups (such as carboxyl, epoxy, hydroxyl, and carbonyl groups), fantastic chemical stability, high strength, superior electron transport, low thickness, high flexibility and a negatively charged surface, innocuity, high surface area, and good miscibility with polymers.^100–113^ The existence of these groups makes GO possess good hydrophilicity, is easy to be modified, and has the capability of dispersion in water to yield a prolonged, stable suspension.^114^ All these factors make it more appropriate for the hydrophilic modification of PES membranes.^115,116^ On this basis, a large amount of work has been devoted to developing GO/PES NC membranes and their summaries are shown in Table 4.

**Table 4:** Progress in recent studies in the fabrication of hydrophilic PES/GO nanocomposite membranes

Additive	Filler loadings (wt%)	Hydrophilic change (°)	Ref.
GO	0.3	72 to ∼55	117
GO/T904	0.3	72 to 54	117
GO NS	0.5	65 to 53	118
GO/PAA	1	∼71 to 58	119
UiO-66@GO	3.0	∼86 to 60	120
GO	3.0	∼86 to 72	120
Partially rGO/TiO_2_	0.1	∼66 to 56	68
GO	0.1	∼66 to ∼59	68
GO–ZnO	0.1	∼78 to ∼54	121
HPEI–GO	5	∼86 to 63	114

### Embedding CNTs

Carbon nanotubes (CNTs) are a member of the fullerene structural family and consist of six-membered carbon rings in the honeycomb lattice relative to the axis of the nanotubes (NT).^122^ The pioneer discovery of CNTs by Lijima^123^ has opened up new directions for many applications. CNTs have the ability to interact and alter the physico-chemical properties of the membrane.^124^ This property coupled with their high specific surface area with low density, exceptional mechanical properties, nanoscale dimensions and highly precise diameters, high thermal stability, very low frictional coefficients on their internal surface, high strength-to weight ratio, formation of highly porous structures, and chemical stability makes CNTs a promising candidate for complementing or substituting conventional NPs in the fabrication of new generation nanocomposite membranes.^125–138^ The excellent mechanical properties of CNTs arise from the presence of C–C bonds in the graphite layer, which are most probably the strongest chemical bonds known in nature. CNTs can be synthesized either as a series of shells of different diameters spaced around a common axis, called multi-walled carbon nanotubes, MWCNTs (consisting of up to 10–100 carbon shells), or as singular tubes, called single-walled carbon nanotubes (SWCNTs).^139^ The former are of particular interest over the latter due to their availability in larger quantities and relatively low cost as a result of their more advanced stage in commercial production.

The most crucial problem when using CNTS is the poor dissolution and dispersion of synthesized CNTs in various organic solvents and different polymers as well as their weak interaction with the polymer matrix.^140–143^ Moreover during CNTs preparation, the presence of metal catalytic particles and amorphous carbon, as impurities, could add an additional burden to the intended application.^144^ These factors are important in the utilization of additives in polymer composites as well as CNTs.^145,146^ Therefore, the purification and functionalization of CNTs could be established to negate the hydrophobic nature of CNTs and to broaden their promising scope. For this reason, different linking groups, *e.g.*, –NH_2_, –SO_3_H, –COOH, –OH, or –CONH_2_ could be introduced to the CNTs surface to facilitate linking different metal clusters to the nanotubes surface *via* polymer wrapping, covalent attachment (grafting), and non-covalent attachment (adsorbing).^122,146–159^ The amine (NH_2_) group has a wealth of chemistry and high reactivity with many chemicals, such as polymers.^160–162^ After modification, they become soluble in different solvents, as well as contain functional groups, which turn them into a multidisciplinary materials in other applications. The functionalization by chemical oxidation of CNTs is the most commonly used method, which breaks the sp^2^ hybrid carbon bonds on the sidewalls, and attaches carboxyl/hydroxyl groups to the CNTs.^163^ Functionalized CNTs can enhance the properties of PES membranes by increasing the hydrophilicity and surface charge of the membrane top layer,^147–151,164,165^ which will influence the permeability and reduce fouling.^147–151,164–169^ An increase in the surface charge will raise the Donnan exclusion effect and electrostatic interactions, which will result in an improved rejection of salt and an increase in hydrophilicity, which will provide better fouling resistance.^170,171^ To date, several authors have shown the successful preparation of CNT-blended PES membranes. The summary of their results are presented in Table 5.

**Table 5:** Progress in recent studies in the fabrication of hydrophilic PES/CNTs nanocomposite membranes

Additive	Treatment	Filler loadings (wt%)	Hydrophilic change (°)	Ref.
CNTs (20 nm)	—	0.1	∼63 to ∼55	172
CNTs (40 nm)	—	0.1	∼63 to 56	172
Carboxyl-functionalized SWCNT	—	0.025	∼70 to ∼62	173
PCA-functionalized MWCNT	*In situ* polymerization reaction	0.1	75 to 49	174
PAA-functionalized MWCNT	*In situ* polymerization reaction	0.1	75 to ∼58	174
SiO_2_	—	3	67 to ∼55	175
Polyacrylamide-functionalized MWCNT	*In situ* polymerization reaction	0.1	75 to ∼63	174
MWCNT	—	0.1	75 to 65	174
NH_2_-MWCNTs	Covalent-functionalization	0.045	∼65 to ∼56	176
PCL modified MWCNTs	With Sn(Oct)_2_	3	∼67 to 57	177
Amine-functionalized MWCNTs	Strong acids (H_2_SO_4_/HNO_3_) and 1,3-phenylenediamine	1	69 to ∼52	1
MWCNT-OH	—	0.8	∼77 to ∼74	178
MWCNT-COOH	—	0.8	∼77 to 59	178
Acid-oxidized MWCNTs	HNO_3_/H_2_SO_4_	0.04	66 to 63	179
MWCNTs	HNO_3_/H_2_SO_4_	2	∼71 to ∼60	168
MWCNTs	—	2	∼65 to ∼47	180
Acid-functionalized MWCNT	PVP	0.1	∼88 to 52	181
Functionalized MWCNT	Non-covalent modification with SLS	2	∼79 to 51	182
Acid-oxidized MWCNTs coated by anatase TiO_2_	Precipitation of TiCl_4_ precursor	0.1	66 to ∼63	183
ZnO coated MWCNTs	Coating	0.5	68 to 57	184
PAA grafted MWCNTs	*In situ* polymerization of AA in aqueous solution in the presence of KPS as initiator and EG as cross-linker	0.1	∼73 to ∼57	185
Ag-coated MWCNTs	Ag	0.9	64 to 51	186
MWCNTs-PANI	*In situ* polymerization in the presence of aniline and APS	2	73 to ∼53	187
Fe–Ag/functionalized MWCNT	Acid and then Fe and Ag NPs	1	75 to ∼44	188
TETA-MWCNTs	—	0.4	∼68 to 60	189
Acid-functionalized MWCNTs	HNO_3_ and H_2_SO_4_	0.5	∼70 to ∼57	190

### Embedding halloysite nanotubes

**Fig. 6:**
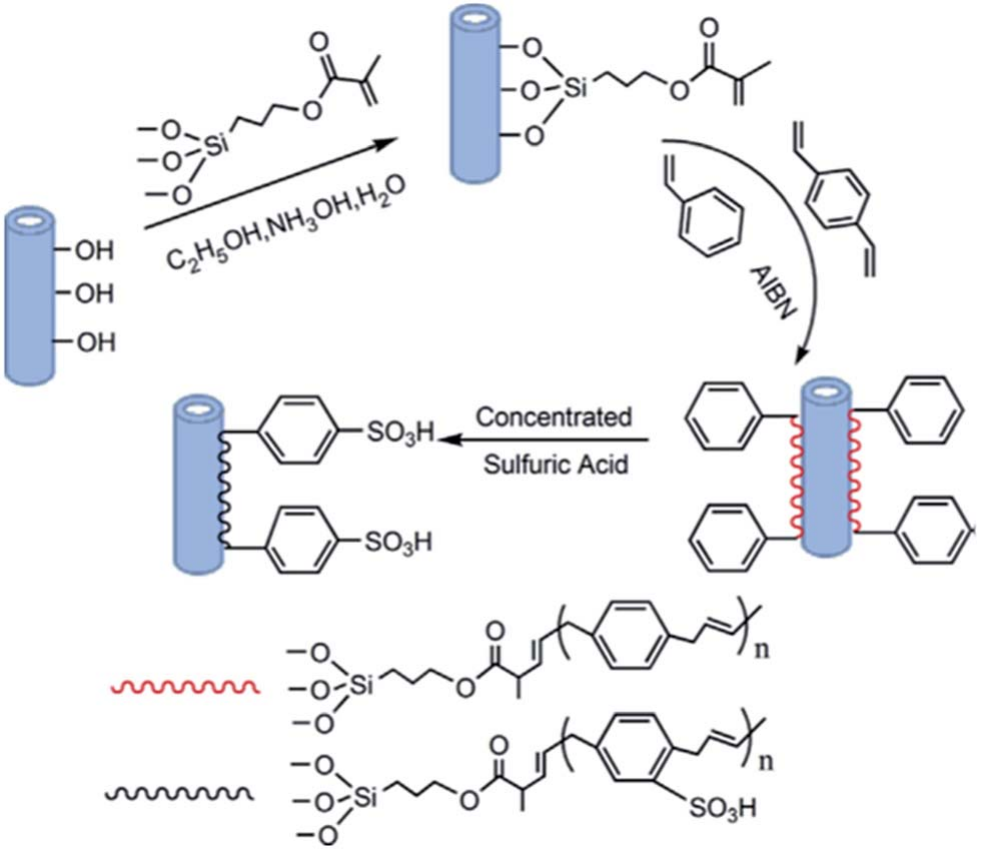
Schematic illustration of the overall preparation process of HNTs-SO_3_H.^193^

Halloysite nanotubes (HNTs) are a kind of naturally occurring aluminosilicate (Al_2_Si_2_O_5_(OH)_4_·2H_2_O) with a hollow nano-tubular structure,^191,192^ regular open-ending pores, as well as a great deal of hydroxyls on their surface.^193^ HNTs can easily be dispersed in a polymer matrix, even at high loading due to their tubular shape, low density of hydroxyl functional groups, and well-crystallized structure.^194–197^ In contrast with other NPs, HNTs can be obtained easily and are much cheaper.^198,199^ HNTs own a low charge density, which means they cannot affect the membrane potential when they are embedded into the polymer matrix.^193^ Recently, HNTs have been used as a new type of filler for PES to improve the properties and performance of the composites. For instance, ref. 200 synthesized a HNTs loaded with copper ions (Cu^2+^-HNTs) by the chemical modification of HNTs, which were then incorporated in the PES matrix to produce Cu^2+^-HNTs/PES MMM, which significantly resulted in an improvement of membrane hydrophilicity, with WCA decreasing from 84.9° to 69.8° for 3 wt% of Cu^2+^-HNTs. Ref. ^193^ presented a sulfonated halloysite nanotubes (HNTs-SO_3_H)/PES membrane. To prepare highly cross-linked HNTs-SO_3_H, styrene was grafted onto HNTs surface *via* distillation–precipitation polymerization and then sulfonated with concentrated sulfuric acid. Fig. 6 shows a schematic illustration of the overall preparation process of HNTs-SO_3_H. The control PES membrane presented the highest contact angle of 83.5°, which was decreased to 58.3° when 3 wt% HNTs-SO_3_H was introduced.

**Fig. 7:**
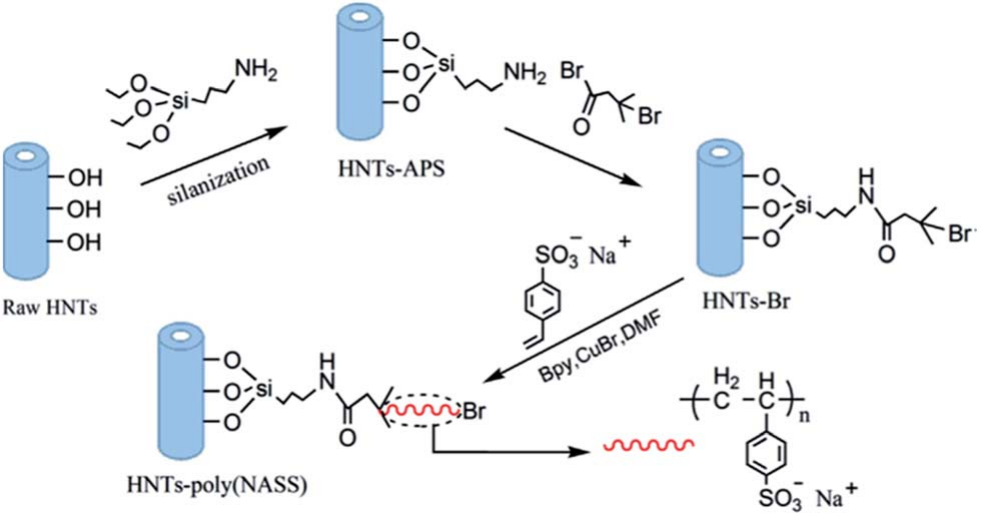
Preparation process of sodium 4-styrene sulfonate grafted onto HNTs surface.^201^

In another study by ref. 201, sodium 4-styrene sulfonate was grafted onto HNTs surfaces *via* surface-initiated atom transfer radical polymerization, as shown in Fig. 7, which was then introduced in the PES matrix to prepared negatively charged nanofiltration membranes. WCA was observed to decrease from 83.5° to 56.6° at 3 wt%.

**Fig. 8:**
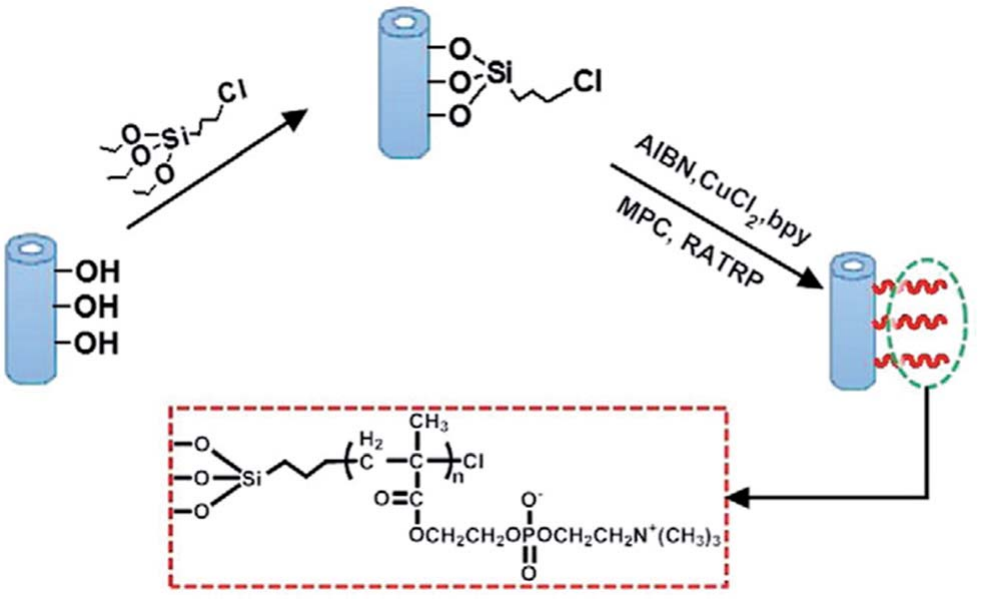
Preparation process of HNTs-MPC *via* reverse atom transfer radical polymerization.^202^

Ref. 202 reported a PES hybrid membrane containing HNTs grafted with 2-methacryloyloxyethyl phosphorylcholine (MPC). Fig. 8 presents the preparation process of HNTs-MPC *via* reverse atom transfer radical polymerization. The contact angle of the membrane decreased with the addition of HNTs-MPC. The WCA value was reduced from 88.4° to 66.1° at 3 wt%.

**Fig. 9:**
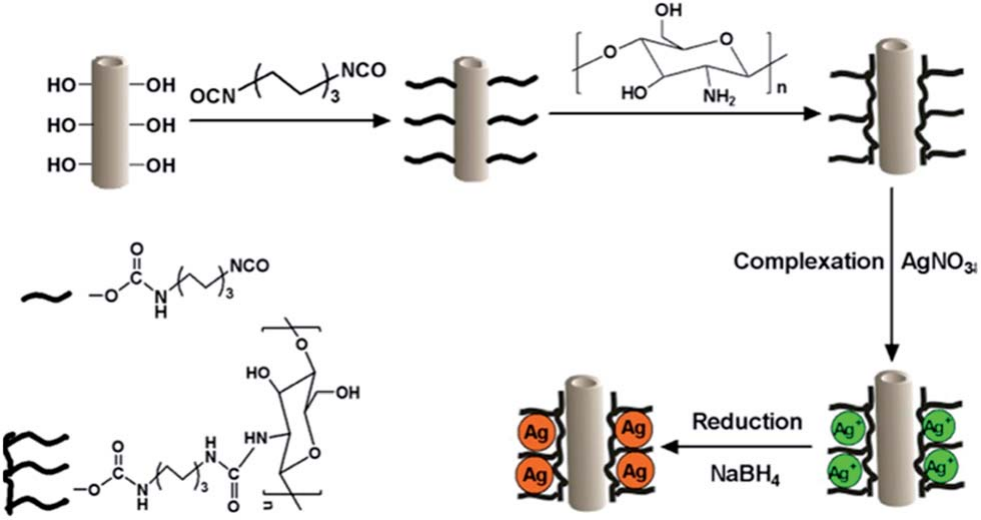
Reaction principle for preparing HNTs-CS@Ag NPs.^203^

Ref. 203 introduced HNTs-chitosan-Ag nanoparticles (HNTs-CS@Ag) into the PES matrix. Prior to blending, the HNTs-CS@Ag were synthesized by chemically modifying HNTs with chitosan, and then mixing with silver nitrate for complexing the silver ions, and finally the silver NPs were formed using sodium tetrahydroborate as a reducing agent. Fig. 9 presents the reaction principle for preparing the HNTs-CS@Ag NPs. The hybrid membranes were shown to be more hydrophilic, with the optimum membrane displaying the lowest contact angle of 55° when the content of HNTs-CS@Ag amounted to 3 wt%.

**Fig. 10:**
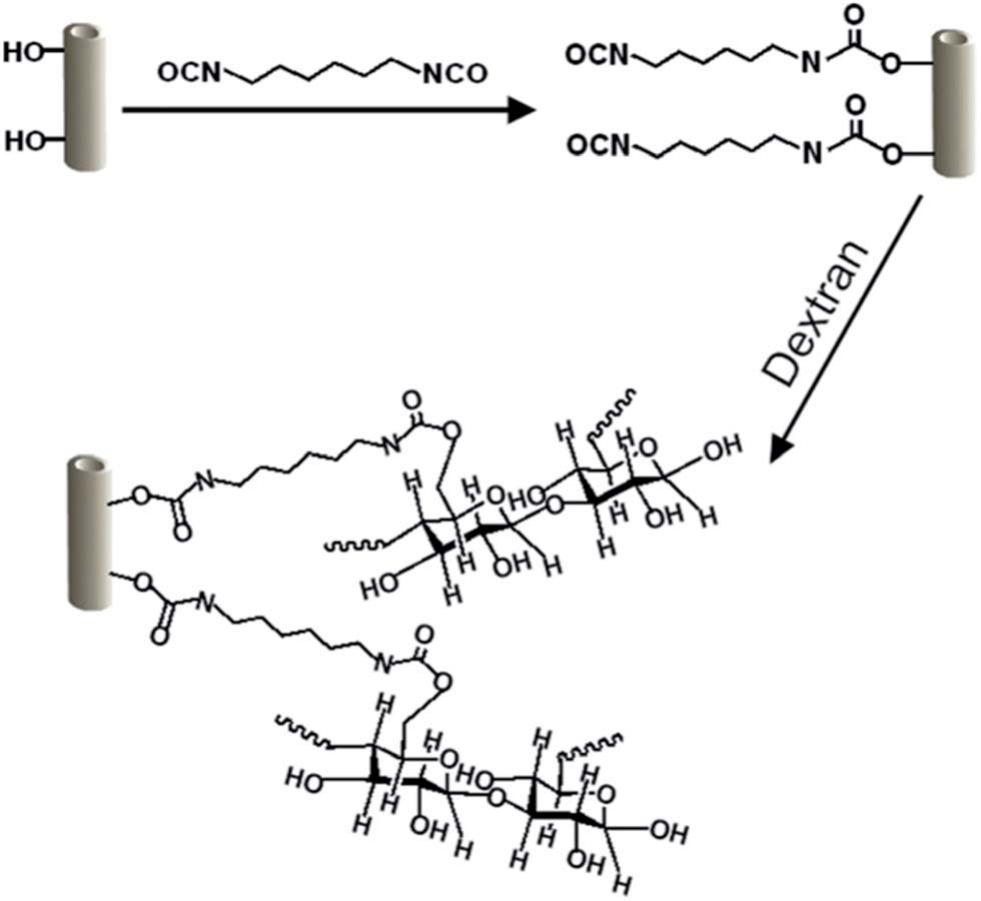
Basic principles of the reactions of the modified HNTs.^204^

Ref. 204 presented polyethersulfone (PES) ultrafiltration membrane by incorporating dextran grafted HNTs (HNTs-dextran). Fig. 10 presents the basic reactions of the modified HNTs. The results indicated that the surface hydrophilicity of the membranes was significantly improved after adding HNTs-dextran. The WCA of the pristine PES membrane amounted to 90.8°, while the WCA of the hybrid membrane with the modified HNTs-dextran content of 3% was 58.3°.

### Embedding metal–organic frameworks

Metal–organic frameworks (MOFs) are organic–inorganic hybrid solids with infinite, uniform framework structures built from inorganic metal (or metal-containing cluster) nodes and organic linkers.^205–208^ MOFs are zeolite-like structures but they do not have the limitations of zeolites in terms of the material's chemistry. MOFs are recently attracting a lot of attention as potential additive materials for MMMs, owing to their extraordinary porosity (as high as 50% of the crystal volume), high surface area (ranging from 1000 to 10 000 m^2^ g^−1^), affinity for certain molecules, capability for functionalization, low density (0.2–1 g cm^−3^), tunable chemical composition, and flexible structure.^209–212^ MOFs have regular and highly harmonic pore structures and they play a very vital role in increasing the hydrophilic property of the membrane.^213–223^

To date, different types of MOFs, including zeolitic imidazolate framework (ZIF), ZIF-8, [Zn(oba)(4-bpdh)_0.5_]·(DMF)_1.5_ (TMU-5), UiO-66, matériauxs de l'Institut Lavoisier, have been developed and introduced in the PES matrix to prepare a MMM. Their potentials have been well recognized both experimentally and computationally.^120,224–230^ The emerging zirconium MOFs (Zr-MOFs) has exhibited exceptionally high chemical and thermal stability.^231^Ref. 224 reported a novel hydrophilic PES/TMU-5 UF membrane synthesized by blending with TMU-5. The growth of TMU-5 upon silk fiber was achieved by sequential dipping in alternating baths of aqueous Zn(NO_3_)_2_·6H_2_O and DMF solution of 4-bpdh and (H_2_oba) under an ultrasound bath. They found that upon the addition of 0.1 wt% NPs, the hydrophilicity was enhanced, with WCA declining from 67.2° to 57.5°. In another study by ref. 225, a two-dimensional zeolitic imidazolate framework with a leaf-shaped morphology (ZIF-L) was synthesized in zinc salt and 2-methylimidazole aqueous solution and then doped in the PES matrix to prepare the MMM. Upon the introduction of 0.5 wt% loading of NPs, the WCA declined slightly to 62.72° as compared to the neat membrane of 67.72°.

### Other inorganic materials

Ref. 232 synthesized hydrous manganese oxide (HMO) from the oxidation of manganous ions by permanganate and then impregnated them in the PES matrix which yielded a WCA of 27.2° at 1.5 wt% of HMO as compared to the nascent membrane of 69.5°. Ref. 233 embedded mesoporous carbon nanoparticles (MCNs) to prepare a hydrophilic PES membrane, which resulted in a decreased WCA from 75° to 36° when the content of MCM was increased to 0.2 wt%. In a study by ref. 20, cellulose nanocrystals (CNCs) were incorporated into PES membranes, which resulted in an improved hydrophilicity from 66.2° to 51.3° at 1 wt% of CNC. In another study by ref. 234, chitosan nanobiopolymers (CS-NPs) were synthesized using tripoly phosphate and introduced in the PES matrix to prepare a nanobiopolymer composite membrane. The addition of 0.2 wt% nanopolymer caused a reduction of WCA from 73° to 65°. Ref. 235 incorporated aragonite precipitated calcium carbonate (A-PCC) from magnesium rich carbonate rock into the PES matrix to prepare a PES composite membrane. They observed a reduction in WCA from 72.3° to 62.5° when the content of the A-PCC was 3 wt%.

### Applications of the hydrophilic PES membrane

With the increasing demand for functional hydrophilic membrane materials, a great deal of attention has been focused on the development of hydrophilic PES membranes. Due to their interaction with water, the use of a hydrophilic PES membrane has found use in various applications, such as desalination, water treatment, wastewater treatment, textile applications, and protein purifications. In fact, hydrophilic PES membrane modification *via* blending is a simple approach to overcoming the performance trade-off and minimizing membrane fouling. A significant number of works have shown that enhancing the hydrophilicity of the PES membrane will result in a reduction of membrane fouling as well as leading to performance improvement. Tables 6 and 7 show summaries of the applications of hydrophilic PES membranes.

**Table 6:** Summaries of the applications of hydrophilic PES-organic membranes

Additive	Application (operating pressure)	Performance of neat membrane {MR (m^−1^); PWF (L m^−2^ h^−1^); PF (L m^−2^ h^−1^); HP (L m^−2^ h^−1^); *R* (%); FRR (%); RFR (%)}	Performance of composite membrane {MR (m^−1^); PWF (L m^−2^ h^−1^); PF (L m^−2^ h^−1^); HP (L m^−2^ h^−1^); *R* (%); FRR (%); RFR (%)}	Ref.
PSA-PVP	Protein purification (4 bar)	PWF: 12.3; PF: 9.5; FRR: 76; BSA *R*: 95.9	PWF: 244.2; PF: 57; FRR: 60.2; BSA *R*: 97.8	27
PA-6	Protein purification (4 bar)	PWF: 7.1; BSA *R*: 92.4; FRR: N/A	PWF: 80.7; BSA *R*: 98.5; FRR: 64.2	26
PEG	Water treatment (1 bar)	HP: 4.998; MR: 8.060 × 10^−11^	HP: 9.422; MR: 4.275 × 10^−11^	22
PVP	Protein purification (4 bar)	PWF: 7.1; BSA *R*: 92.4; FRR: (N/A)	PWF: 166.5; BSA *R*: 95.6; FRR: 57	26
PVP	Protein purification (4 bar)	PWF: 12.3; PF: 9.5; FRR: 76; BSA *R*: 95.9	PWF: 277.4; PF: 63.6; FRR: 54.6; BSA *R*: 97.6	27
PEG-PVP	Water treatment (2 bar)	PWF: 2201.8	PWF: 18 899.1	23
PES-*g*-PDMAEMA	Protein purification (2 bar)	PWF: 18.76; *R*: 99	PWF: 126.7; *R*: 96.4	33
PEG	Protein purification (1 bar)	PWF: N/A; BSA *R*: N/A	PWF: 76.7; BSA *R*: 99	236
PVP-*b*-PMMA-*b*-PVP	Protein purification (0.05 bar)	FRR: 50.6; PA: 19.3	FRR: 96.6; PA: 10	39
PVP	Water treatment (1 bar)	PWF: 64.34; *R*: 96.03	PWF: 108.09; *R*: 88.07	24
PES-*g*-PNIPAAm	Protein purification (2 bar)	PWF: 18.76; *R*: 99	PWF: 110; *R*: 97.5	33
PVP	Wastewater treatment (1 bar)	PWF: 2.2; BSA *R*: 88.9	PWF: 15.8; BSA *R*: 65	25
PES-*b*-PSBMA	Protein purification (1 bar)	PWF: 45.6; FRR: 49.3; BSA *R*: 76	PWF: 115; FRR: 84.2; BSA *R*: 80.7	40
P31R1	Wastewater treatment (1 bar)	PWF: 2.2; BSA *R*: 88.9	PWF: 116.5; BSA *R*: 62.4	25
F127-*b*-PDMAEMA	Protein purification (2 bar)	PA: 13.2	PA: 34.3	34
T904	Wastewater treatment (1 bar)	PWF: 2.2; BSA *R*: 88.9	PWF: 62; BSA *R*: 69.2	25
PVP	Protein purification (2.5 bar)	PWF: 67.6; PEG_35k_*R*: 99.2; PEG_20k_*R*: 98.1; PEG_10k_*R*: 97.9; PEG_4k_*R*: 93.5	PWF: 134.8; PEG_35k_*R*: 97.9; PEG_20k_*R*: 97.9; PEG_10k_*R*: 97.3; PEG_4k_*R*: 94.8	237
PSf-*g*-POEM	Protein purification (1.5 bar)	PA: 44.2; BSA *R*: 74; FRR: 51.7	PA: 22.3; BSA *R*: 0; FRR: 80.8	35
PVP	Wastewater treatment (5 bar)	PWF: 108.21; FRR: 38.8; COD *R*: 79.4; TDS *R*: 71.3	PWF: 59.2; FRR: 59; COD *R*: 84.4; TDS *R*: 78.5	28
PVP	Water treatment (2 bar)	PWF: N/A; PF: N/A; FRR: N/A; HA *R*: N/A	PWF: 2439; PF: 266.5; FRR: 98.5; HA *R*: 89.4	238
MF-*g*-PEG_6k_	Water treatment (1 bar)	PWF: 60.7; FRR: 70.8	PWF: 164.7; FRR: 91.6	37
PVP	Water treatment (0.2–0.3 bar)	PWF: 128.26	PWF: 376.8	239
PMAAn–F127–PMAA	Protein purification (1 bar)	PWF: 180.8; BSA *R*: 96.75	PWF: 238.6; BSA *R*: 85.5	38
NPhthCs	Water treatment (3–6 bar)	BSA flux: 17.6; HP: 7.1; BSA *R*: 90	BSA flux: 55.2; HP: 26.8; BSA *R*: 86	29

**Table 7:** Summaries of the applications of hydrophilic PES–inorganic membranes

Additives	Applications (operating pressure)	Performance of neat membrane {PWF (L m^−2^ h^−1^); PF (L m^−2^ h^−1^); *R* (%); FRR (%); RFR (%)}	Performance of composite membrane {PWF (L m^−2^ h^−1^); PF (L m^−2^ h^−1^); *R* (%); FRR (%); RFR (%)}	Ref.
CNTs	Desalination (4 bar)	PWF: 24.25; Na_2_SO_4_*R*: 24.7	PWF: 52.86; Na_2_SO_4_*R*: 71.71	172
Fe_3_O_4_	Water treatment (4 bar)	Cu^+^*R*: 19.5; PWF: 8.8	Cu^+^*R*: 34.4; PWF: 16.8	92
CNTs	Desalination (4 bar)	PWF: 24.25; Na_2_SO_4_*R*: 24.7	PWF: 38.91; Na_2_SO_4_*R*: 87.25	172
TiO_2_	Water treatment (10 bar)	PWF: 1.7	PWF: 8.2	66
Fe_3_O_4_–SiO_2_	Water treatment (4 bar)	Cu^+^*R*: 19.5; PWF: 8.8	Cu^+^*R*: 40.7; PWF: 32.6	92
GO	Protein purification (1 bar)	PWF: 2; FRR: 26; BSA *R*: 88.6	PWF: 37; FRR: 58; BSA *R*: 95.3	117
TiO_2_	Wastewater treatment (6 bar)	PWF: 21; DR23 *R*: 97	PWF: 33.4; DR23 *R*: 94.9	67
SWCNT	Wastewater treatment (2 bar)	FRR: 93.7; BPA *R*: 45.7; NPH *R*: 62.95; BPA flux: 30.5; NPH flux: 42	FRR: 96.8; BPA *R*: 45.2; NPH *R*: 59.2; BPA flux: 30.2; NPH flux: 39.5	173
PES/Fe_3_O_4_–SiO_2_-amine	Water treatment (4 bar)	Cu^+^*R*: 19.5; PWF: 8.8	Cu^+^*R*: 79.7; PWF: 14.3	92
TiO_2_	Wastewater treatment (5 bar)	PWF: 23; FRR: 75.2; RG19 *R*: 92.9; RB21 *R*: 88.9; DY12 *R*: 61.4	PWF: 32.6; FRR: 87.4; RG19 *R*: 99; RB21 *R*: 73.2; DY12 *R*: 91.1	68
MWCNT	Water treatment (4 bar)	FRR: 44; PWF: 9	FRR: 95; PWF: 23	174
Fe_3_O_4_–SiO_2_-Met	Water treatment (4 bar)	Cu^+^*R*: 19.5; PWF: 8.8	Cu^+^*R*: 92.3; PWF: 27.8	92
Mechanically modified TiO_2_	Water treatment (1 bar)	FRR: 83.33; PWF: 17.6	FRR: 51.85; PWF: 39.8	71
FeN	Water treatment (1 bar)	PWF: 6.1; Cu + *R*: 95; Zn^2+^*R*: 95; Cu^+^ flux: 4.7; Zn^2+^ flux: 5.2	PWF: 24; Cu + *R*: 89.7; Zn^2+^*R*: 87; Cu^+^ flux: 12.9; Zn^2+^ flux: 13.6	91
MWCNT	Water treatment (4 bar)	FRR: 44; PWF: 9	FRR: 67; PWF: 30.5	174
Mechanically and chemically modified TiO_2_	Water treatment (1 bar)	FRR: 83.33; PWF: 17.6	FRR: 61.54; PWF: 54.9	71
GO-T904	Protein purification (1 bar)	PWF: 2; FRR: 26; BSA *R*: 89.3	PWF: 245; FRR: 62; BSA *R*: 93.6	117
A-PCC	Wastewater treatment (1.5 bar)	PWF: 102; PF: 49.87; FRR: 61.8; oil *R*: 93.9	PWF: 180; PF: 102.15; FRR: 86.4; oil *R*: 99.8	235
Fe_3_O_4_	Water treatment (1–10 bar)	PWF: 12.3; NaCl *R*: 15.3; MgSO_4_*R*: 16.16	PWF: 86.2; NaCl *R*: 68; MgSO_4_*R*: 82	83
Mechanically and chemically modified TiO_2_	Water treatment (1 bar)	FRR: 60; PA: 33.5; PWF: 364.8	FRR: 84; PA: 22.6; PWF: 462.3	69
MWCNT	Water treatment (4 bar)	FRR: 44; PWF: 9	FRR: 76; PWF: 26	174
*n*-Ag	Protein purification (1 bar)	PWF: 25; FRR: 53.5; PF: 42.7; BSA *R*: 97.4	PWF: 64; FRR: 79.4; PF: 67.7; BSA *R*: 90.3	99
TiO_2_	Water treatment (1 bar)	FRR: 60; PA: 33.5; PWF: 364.8	FRR: 57; PA: 37.5; PWF: 345.9	69
MWCNT	Water treatment (4 bar)	FRR: 44; PWF: 9	FRR: 53; PWF: 23	174
AgNP	Water treatment (2–4 bar)	PWF: 365	PWF: 327	97
SiO_2_@N-Halamine	Water treatment (1 bar)	PVA *R*: 96.2; PWF: 192.7; FRR: 87	PVA *R*: 94.8; PWF: 384.9; FRR: 96	77
Amine-functionalized MWCNT	Desalination (4 bar)	PWF: 13.6; Na_2_SO_4_*R*: 52; FRR: 68.6; PA: 60.9	PWF: 23.7; Na_2_SO_4_*R*: 65; FRR: 88.1; PA: 41	176
TMU-5	Water treatment (3 bar)	PWF: 133.29; FRR: 24.47	PWF: 182.02; FRR: 98.74	224
Mesostructured SBA-15	Water treatment (3 bar)	PWF: 181.4	PWF: 316.1	75
PCL modified CNT	Desalination (8 bar)	PWF: 28; Cd ions *R*: 8.7; FRR: 13.3; BSA flux: 4.3	PWF: 61; Cd ions *R*: 27; FRR: 11.1; BSA flux: 6.4	177
PES/mesostructured SBA-15/CES-15	Water treatment (3 bar)	PWF: 181.4	PWF: 351.7	75
GO	Wastewater treatment (4 bar)	PWF: 8.2; FRR: 35; dye *R*: 90	PWF: 20.4; FRR: 90.5; dye *R*: 96	118
Mesostructured SBA-15-APTMS-15	Water treatment (3 bar)	PWF: 181.4	PWF: 356.8	75
ZIF-L	Water treatment (1 bar)	PWF: 215; FRR: 72	PWF: 378; FRR: 82	225
F-MWCNTs	Protein purification (3 bar)	PWF: 124; PF: 14.5; BSA *R*: 86.5; FRR: 27	PWF: 184; PF: 33.2; BSA *R*: 81; FRR: 46	1
Mesostructured SBA-15/AEAPTMS-15	Water treatment (3 bar)	PWF: 181.4	PWF: 595.8	75
Hydroxylated MWCNT	Water treatment (1 bar)	PWF: 587.1	PWF: 812.9	178
Mesostructured SBA-15/CES-30	Water treatment (3 bar)	PWF: 181.4	PWF: 463.6	75
Carboxylated MWCNT	Water treatment (1 bar)	PWF: 587.1	PWF: 412.9	178
Nano-SiO_2_	Wastewater treatment (2–6 bar)	FRR: 82.1; HA flux: 59.2; HA *R*: 94.1; MB flux: 81.1; MB *R*: 32.5	FRR: 86.2; HA flux: 77.4; HA *R*: 94.7; MB flux: 92.9; MB *R*: 42.2	74
GO-PAA	Wastewater treatment (4 bar)	PWF: 43; PF: 9.8; CR (SM): 44.1; CR (SWE): 39.7	PWF: 57; PF: 21.8; CR (SM): 53.5; CR (SWE): 48.8	119
SiO_2_	Water treatment (4 bar)	PWF: 249.37; BSA *R*: 91.9; FRR: 80.6	PWF: 510.76; BSA *R*: 97.8; FRR: 98	73
Oxidized MWCNT	Desalination (4 bar)	PWF: 5; Na_2_SO_4_*R*: 20; FRR: 29.7	PWF: 7.3; Na_2_SO_4_*R*: 75; FRR: 87.7	179
ZnO	Protein purification (1 bar)	PWF: 32.8; BSA *R*: 99.2; RFR: 27.7	PWF: 116.6; BSA *R*: 98.8; RFR: 7.8	82
CNT	Water treatment (4.1 bar)	TOC *R*: 35.5; UVA_254_*R*: 20.2	TOC *R*: 48.8; UVA_254_*R*: 41.8	168
ZnO	Water treatment (1.5 bar)	FRR: 97.01; PWF: 30.42; HA flux: 24.84; RFR: 17.72	FRR: 91.1; PWF: 51.01; HA flux: 44.64; RFR: 12.27	81
CNT	Water treatment (3.5 bar)	BSA *R*: 95.8; OVA *R*: 95.2; BSA flux: 3.2; OVA flux: 2.1; FRR (OVA*): 31.7; FRR (BSA): 40.8; PWF: 12	BSA *R*: 98.2; OVA *R*: 98.1; BSA flux: 35.1; OVA flux: 26.1; FRR (OVA*): 70.83; FRR (BSA): 80; PWF: 70	180
ZnO	Water treatment (4 bar)	PWF: 31; FRR: 39.4	PWF: 48; FRR: 68.9	84
UiO-66@GO	Water treatment (2.5 bar)	PWF: 3.8; DR *R*: 93.1; MO *R*: 85; FRR: 42.9	PWF: 15.8; DR *R*: 98.4; MO *R*: 89.1; FRR: 88.6	120
ZnO nanorod	Water treatment (4 bar)	PWF: 31; FRR: 39.4	PWF: 50; FRR: 73.1	84
Nano-hybrid f-MWCNT/PVP_90_	Protein purification (2.75–3.25 bar)	PWF: 7.6; PA: 16.9	PWF: 71.7; PA: 7	181
CS-ZnO HNPS	Water treatment (N/A)	PWF: 1215.8	PWF: 4135.8	85
SLS-CNT	Protein purification (1 bar)	PWF: 141; BSA *R*: 97.9; PA: 111.4; FRR: 61.4	PWF: 595.6; BSA *R*: 95.8; PA: 56.9; FRR: 94.4	182
CZN	Protein purification (3 bar)	BSA flux: 92.5; FRR: 44.6; PWF: 514.1	BSA flux: 117.7; FRR: 50.1; PWF: 678.5	86
TiO_2_ coated MWCNT	Desalination (5 bar)	PWF: 3.71; FRR: 53.1; Na_2_SO_4_*R*: 69.5; NaCl *R*: 36.1	PWF: 4.35; FRR: 83; Na_2_SO_4_*R*: 80.7; NaCl *R*: 41.4	183
ZrO_2_	Wastewater treatment (0.345–3.1 bar)	PWF: 878.3	PWF: 1581	240
GO	Water treatment (at 2.5 bar)	PWF: 3.8; DR *R*: 93.1; MO *R*: 85; FRR: 42.9	PWF: 8.8; DR *R*: 87.9; MO *R*: 81.4; FRR: 84.3	120
ZrO_2_	Protein purification (1 bar)	BSA *R*: 97.2; OVA *R*: 94.6; PWF: 8.2	BSA *R*: 92.7; OVA *R*: 91.2; PWF: 83.6	89
ZnO coated MWCNTs	Wastewater treatment (4 bar)	Dye *R*: 91; PWF: 8.2; PF: 7.5; FRR: 67.1	Dye *R*: 96.1; PWF: 16.7; PF: 16.1; FRR: 95.2	184
Al_2_O_3_	Water treatment (4.5 bar)	PWF: 8.5; Cu *R*: 28	PWF: 25.3; Cu *R*: 55.9	90
PAA grafted MWCNTs	Desalination (4 bar)	Na_2_SO_4_*R*: 48.4; NaCl *R*: 0.56; PWF: 8.9; FRR: 52	Na_2_SO_4_*R*: 65.5; NaCl *R*: 19.1; PWF: 29.3; FRR: 69	185
Alumina	Protein purification (2 bar)	PWF: 182.2; BSA *R*: 98.8	PWF: 209; BSA *R*: 96.7	70
rGO-TiO_2_	Wastewater treatment (5 bar)	PWF: 23; FRR: 75.2; RG19 *R*: 92.9; DY12 *R*: 88.9; RB21 *R*: 61.4	PWF: 43.3; FRR: 96.8; RG19 *R*: 99.2; DY12 *R*: 95.2; RB21 *R*: 81.4	68
ZrO_2_	Protein purification (2 bar)	PWF: 182.2; BSA *R*: 98.8	PWF: 190.1; BSA *R*: 95.2	70
Ag coated MWCNTs	Water treatment (4 bar)	PWF: 554	PWF: 556	186
TiO_2_	Protein purification (2 bar)	PWF: 182.2; BSA *R*: 98.8	PWF: 198.6; BSA *R*: 95.5	70
MWCNTs-PANI	Water treatment (1 bar)	HA *R*: 18.4; PWF: 265.4	HA *R*: 62.9; PWF: 1498.1	187
Al_2_O_3_	Water treatment (0.69–1.03 bar)	PWF: 866.5	PWF: 1268	241,242
HMO(0.75)–TiO_2_ (0.25)	Wastewater treatment (1 bar)	PWF: 23.71; oil *R*: 98.16; FRR: 45.9	PWF: 28.48; oil *R*: 98.57; FRR: 91.5	243
GO	Wastewater treatment (5 bar)	PWF: 23; FRR: 75.2; RG19 *R*: 92.9; DY12 *R*: 88.9; RB21 *R*: 61.4	PWF: 35.9; FRR: 90.7; RG19 *R*: 98.7; DY12 *R*: 89.6; RB21 *R*: 69.4	68
Cu2+-HNTs	Protein purification (1 bar)	PF: 73.1; PEG_10k_*R*: 84.3; PEG_20k_*R*: 95.2	PF: 120; PEG_10k_*R*: 73.9; PEG_20k_*R*: 93	200
Magnetic-treated Fe_3_O_4_	Water treatment (4 bar)	PWF: 36; FRR: 52	PWF: 65; FRR: 77.7	93
Fe–Ag/f-MWCNT	Water treatment (4 bar)	PWF: 26.5; Cr^6+^ ions *R*: 9.34; FRR: 64	PWF: 36.9; Cr^6+^ ions *R*: 94.8; FRR: 94.98	188
HMO(0.25)–TiO_2_ (0.75)	Wastewater treatment (1 bar)	PWF: 23.71; *R*: 98.16; FRR: 45.9	PWF: 27.33; *R*: 97.17; FRR: 63.6	243
HMO	Protein purification (1.5 bar)	PWF: 39.4; BSA *R*: 57; pepsin *R*: 50; trypsin *R*: 39; FRR: 53.7; BSA flux: 17.3	PWF: 499.2; BSA *R*: 85; pepsin *R*: 70; trypsin *R*: 66; FRR: 96.2; BSA flux: 158.4	232
SiO_2_	Wastewater treatment (1.5 bar)	PWF: N/A; PF: N/A; oil *R*: N/A; FRR: N/A	PWF: 117; PF: 76.67; oil *R*: 98.57; FRR: 81	244
Sulfonated HNT	Desalination and wastewater treatment (4 bar)	PWF: 29.3; NaCl *R*: 16.5; MgCl_2_*R*: 22.8; Na_2_SO_4_*R*: 17.2; MgSO_4_*R*: 17.5; RR49 *R*: 93.6; RB5 *R*: 94.9	PWF: 40.3; NaCl *R*: 1.9; MgCl_2_*R*: 7.4; Na_2_SO_4_*R*: 16.4; MgSO_4_*R*: 13.6; RR49 *R*: 90.4; RB5 *R*: 94	193
Magnetic-treated PANI/Fe_3_O_4_	Water treatment (4 bar)	PWF: 36; FRR: 52	PWF: 52; FRR: 80	93
GO-ZnO	Wastewater treatment (5 bar)	PWF: 1.5; salt *R*: 17	PWF: 13.5; salt *R*: 28	121
Magnetic-treated MWCNT	Water treatment (4 bar)	PWF: 36; FRR: 52	PWF: 29; FRR: 64.6	93
TETA-MWCNT	Desalination (10 bar)	PWF: 36.27; NaCl *R*: 18.58; Na_2_SO_4_*R*: 72.65; MgCl_2_*R*: 47.23; MgSO_4_*R*: 62.08; FRR: 72.9	PWF: 84.35; NaCl *R*: 27.02; Na_2_SO_4_*R*: 32.56; MgCl_2_*R*: 92.73; MgSO_4_*R*: 55.36; FRR: 93.1	189
Chitosan nano-biopolymers	Water treatment (4 bar)	PWF: 13	PWF: 22	234
Fe_3_O_4_	Water treatment (4 bar)	PWF: 36; FRR: 52	PWF: 33; FRR: 68	93
Hollow mesoporous SiO_2_ spheres	Protein purification (1 bar)	PWF: 38; BSA *R*: 93.2; FRR: 62.2	PWF: 195.7; BSA *R*: 92.6; FRR: 82.4	76
HPEI-GO	Protein purification (1 bar)	FRR: 86.6; PWF: 204.5; PA: 61.11	FRR: 92.1; PWF: 206.9; PA: 25.89	114
HNTs-poly(NASS)	Desalination (4 bar)	PWF: 29.4; RR49 *R*: 93.7; RB5 *R*: 95.2; NaCl *R*: 83.5; MgCl_2_*R*: 77; Na_2_SO_4_*R*: 82.8; MgSO_4_*R*: 82.5	PWF: 97.5; RR49 *R*: 90.5; RB5 *R*: 91.7; NaCl *R*: 97.2; MgCl_2_*R*: 96.5; Na_2_SO_4_*R*: 90; MgSO_4_*R*: 90.9	201
HNTs-CS@Ag	Protein purification (1 bar)	PEG_20k_*R*: 94; PWF: 112.11	PEG_20k_*R*: 72.8; PWF: 375.8	203
PANI/Fe_3_O_4_	Protein purification (4 bar)	PWF: 36; FRR: 52	PWF: 33; FRR: 68	93
MCNs	Protein purification (1 bar)	PWF: 218.9; PF: 37.8; FRR: 64.9: PA: 40.3: BSA *R*: 99.9	PWF: 257.8; PF: 30.4; FRR: 60.9: PA: 7.8: BSA *R*: 99.9	233
HNTs-dextran	Protein purification (1 bar)	PWF: 80.3; FRR: 86; PEG_20k_*R*: 93.2; PVA_30–70k_*R*: 96.9	PWF: 224.5; FRR: 96; PEG_20k_*R*: 76.1; PVA_30–70k_*R*: 100	204
f-MWCNTs	Wastewater treatment (4.1 bar)	PWF: 24.28; PF: 4.4	PWF: 53.91; PF: 7.4	190
Fe_3_O_4_-MWCNT	Water treatment (4 bar)	PWF: 36; FRR: 52	PWF: 45; FRR: 76.6	93
HNTs-MPC	Protein purification (1 bar)	FRR: 85.2; PWF: 110.06; PEG_20k_*R*: 91.8; PA: 63.6	FRR: 93.1; PWF: 224.39; PEG_20k_*R*: 83.6; PA: 8.3	202
CNC	Protein purification (2.7 bar)	PWF: 93.4; BSA *R*: 93; FRR: 51	PWF: 195; BSA *R*: 96; FRR: 76.2	20
SiO_2_	Wastewater treatment (1.5 bar)	PWF: 87.347; PF: 60.112; oil *R*: 95.77; FRR: 71.17; RFR: 31.18	PWF: 102.43; PF: 90.937; oil *R*: 99.98; FRR: 93.33; RFR: 11.22	175

## Conclusions and future prospects

Seemingly the permanent hydrophilic modification of PES membranes can be achieved by blending with organic and/or inorganic materials. Furthermore, there is no denying the fact that the amount of data available today on the hydrophilic enhancement of PES membranes *via* blending is a stepping stone to upgrading PES membranes to new heights. Some of the conclusions drawn from this comprehensive review are listed as follows:

• To achieve an improved surface hydrophilicity and performance, many factors need to be considered in the overall process of composite membrane preparation, such as precise control over the functional groups, uniformity, and reproducibility. For instance, the functional groups on CNTs have the ability to be converted to membrane functional groups and can change the surface hydrophilicity and performance of the PES membrane. Therefore, more functional groups on CNTs are expected to reveal more significant changes in membrane hydrophilicity and performance. However, there is also a need for comprehensive investigation concerning the use and influence of multiple-modified SWCNTs and MWCNTs on PES NC membranes characteristics to verify the efficiency of PES modification of CNTs on the surface hydrophilization of PES membranes. Furthermore, the production costs of carbon nanotubes are quite high. Thus, further work should investigate and address the economic aspects so that their potentials for commercial scale can be realized.

• In the case of blending with inorganic materials, the interaction between PES and NPs is specific and the final membrane hydrophilicity and performance depends on such interaction. Therefore, the effectiveness of hydrophilicity will depend on the location of NPs in the membrane matrix because the location of NPs can change the diffusivity in the PES matrix. The surface energy and concentration are other important factors that can affect NPs dispersion and location, which could lead to NPs aggregation on the surface of the PES NC membrane. NPs aggregation will mean that the effectiveness of surface hydropilicity will be reduced during intended applications. To decrease the surface energy or improve the dispersion of NPs in the PES matrix, the surface modification of the NPs by grafting with a polymer can be an effective method.

• The use of a variety of functional and synthetic materials (*i.e.*, lyotropic liquid crystals, aquaporins) will improve the hydrophilicity, enable the highest permeation rates, as well as keep the doors open for research and development in the field of multifunctional, high-performance, and antifouling PES membranes.

• Although, the combination of two or three additives can be more complex in terms of the environmental drawbacks and cost effectiveness, these could lead to multifunctional PES membranes that are of great interest for ‘future hydrophilic PES membranes’. Comparison with the existing ones to determine their adaptability and sustainability for commercial purposes will be the next step.

• With a hydrophilic PES membrane, it should be mentioned that solute adsorption is reduced at the produced hydrophilic surfaces, but is never completely prevented. Therefore, it is expected that membrane surface hydrophilicity can be tuned for specific applications through the discussed methods, although they still need to be developed further in such a way that they allow even more and better environmentally friendly control over other modification methods.

• Finally, but also very important, is the processing ability and economic cost. Generally, the cost is a major concern in the commercialization of membrane technology. Some hydrophilic PES membranes might produce a better quality of permeate and solute removal but the operating costs may be higher. Thus, the cost associated with the synthesis and incorporation of these additives needs to be addressed at the earliest for their development from the laboratory to a commercial-applicable scale.

## Conflicts of interest

We declare that there is no conflict of interest in this work.

## Symbols and abbreviations

AA: Acrylic acid
AEAPTMS: [3-(2-Aminoethylamino)propyl] trimethoxysilane
Ag: Silver
Al_2_O_3_, AgNP: Silver nanoparticles
Al_2_O_3_: Aluminum oxide
A-PCC: Aragonite precipitated calcium carbonate
APS: Ammonium peroxidisulfate
APTMS: (3-Aminopropyl) trimethoxysilane
ATRP: Atom transfer radical polymerization
4-bpdh: 2,5-Bis(4-pyridyl)-3,4-diaza-2,4-hexadiene
BPA: Bisphenol A
BSA: Bovine serum albumin
CES: Carboxyethylsilanetriol sodium salt
COD: Chemical oxygen demand
COOH: Carboxyl
CNC: Cellulose nanocrystals
CNTs: Carbon nanotubes
CR: Color rejection
CS: Chitosan
Cu^2+^: Copper ions
CZN: CuO/ZnO nanocomposite
DIW: Deionized water
DR: Direct red 80
DY12: Direct yellow 12
EG: Ethylene glycol
F127-*b*-PDMAEMA: F127-based amphiphilic *block* copolymers containing poly(*N*,*N*-dimethylamino-2-ethyl methacrylate) end blocks
Fe-NPs: Iron oxide-based nanoparticles
FeN: Iron nanoparticles
FRR (%): Flux recovery ratio
GO: Graphene oxide
H_2_oba: 4,4′-Oxybisbenzoic acid
HA: Humic acid
HMSS: Hollow mesoporous silica sphere
HNTs: Halloysite nanotubes
HNTs-SO_3_H: Sulfonated halloysite nanotubes
HP (L m^−2^ h^−1^): Hydraulic permeability
HPEI: Hyperbranched polyethylenimine
KPS: Potassium persulfate
MB: Methylene blue
Met: Metformine
MF: Melamine formaldehyde
MgO: Manganese oxide
MO: Methyl orange
MOFs: Metal–organic frameworks
m-Fe_3_O_4_: Magnetic-treated Fe_3_O_4_
m-PANI/Fe_3_O_4_: Magnetic-treated polyaniline-coated Fe_3_O_4_
m-MWCNT/Fe_3_O_4_: Magnetic-treated Fe_3_O_4_ coated multi-walled carbon nanotubes
MMM: Mixed matrix membrane
MPC: 2-Methacryloyloxyethyl phosphorylcholine
MR (m^−1^): Membrane resistance
NC: Nanocomposite
NPH: 4-Nonylphenol
NPhthCs: *N*-Phthaloyl-chitosan
NPs: Nanoparticles
NT: Nanotubes
OH: Hydroxyl
OVA: Ovalbumin
P31R1: Linear pluronic 31R1
PA: Protein adsorption
PA-6: Polyamide
PAA: Polyacrylic acid
PANI/Fe_3_O_4_: Polyaniline-coated Fe_3_O_4_
PCA: Polycitric acid
PCL: Polycaprolactone
PDMAEMA: Poly(*N*,*N*-dimethylamino-2-ethyl methacrylate)
PEG: Polyethylene glycol
PEO: Polyethylene oxide
PES: Polyethersulfone
PES-*g*-PSBMA: Polyethersulfone-*block*-poly(sulfobetaine methacrylate)
PF (L m^−2^ h^−1^): Permeate flux
PMAA: Poly(methacrylic acid)
PNIPAAm: Poly (*N*-isopropyl acrylamino)
PS-*b*-PAA: Polystyrene-*block*-poly(acrylic acid)
PSA: Polysulfide-amide
PSf-*g*-POEM: polysulfone-*graft*-poly (ethylene glycol) methyl ether methacrylate
PVP: Polyvinyl pyrrolidone
PWF (L m^−2^ h^−1^): Pure water flux
R (%): Rejection
RAFT: Reversible addition-fragmentation polymerization
RB5: Reactive black 5
RB 21: Reactive blue 21
RFR (%): Reversible fouling ratio
RG 19: Reactive green 19
rGO: Partially reduced graphene oxide
RR49: Reactive red 49
SiO_2_: Silicon dioxide
SLS: Sodium lignosulfonate
SM: Synthetic melanoidin
SWCNTs: Single-walled carbon nanotubes
SWE: Spent wash effluent
T904Star-like tetronic: Star-like tetronic 904
TDS: Total dissolved solids
TEOS: Tetraethyl orthosilicate
TETA: Triethylenetetramine
TiO_2_: Titanium dioxide
TMU-5: [Zn(oba)(4-bpdh)_0.5_]·(DMF)_1.5_
WCA: Water contact angle
WF (L m^−2^ h^−1^): Water flux
ZIF: Zeolitic imidazolate framework
ZIF-L: Zeolitic imidazole framework with leaf-shaped morphology (ZIF-L)
ZnO: Zinc oxide
ZrO_2_: Zirconium dioxide

## Supplementary Material
